# Poverty identification for a pro-poor health insurance scheme in Tanzania: reliability and multi-level stakeholder perceptions

**DOI:** 10.1186/s12939-015-0273-9

**Published:** 2015-12-01

**Authors:** August Kuwawenaruwa, Jitihada Baraka, Kate Ramsey, Fatuma Manzi, Ben Bellows, Josephine Borghi

**Affiliations:** Ifakara Health Institute, Plot 463, Kiko Avenue Mikocheni, P.O. Box 78 373, Dar es Salaam, Tanzania; Department of Global Health and Development, London School of Hygiene & Tropical Medicine, London, UK; Columbia University, Mailman School of Public Health, New York, NY USA; Population Council, Lusaka, Zambia

**Keywords:** Poverty identification, Insurance, Wealth index, Consumption expenditure, Tanzania

## Abstract

**Background:**

Many low income countries have policies to exempt the poor from user charges in public facilities. Reliably identifying the poor is a challenge when implementing such policies. In Tanzania, a scorecard system was established in 2011, within a programme providing free national health insurance fund (NHIF) cards, to identify poor pregnant women and their families, based on eight components. Using a series of reliability tests on a 2012 dataset of 2,621 households in two districts, this study compares household poverty levels using the scorecard, a wealth index, and monthly consumption expenditures.

**Methods:**

We compared the distributions of the three wealth measures, and the consistency of household poverty classification using cross-tabulations and the Kappa statistic. We measured errors of inclusion and exclusion of the scorecard relative to the other methods. We also gathered perceptions of the scorecard criteria through qualitative interviews with stakeholders at multiple levels of the health system.

**Findings:**

The distribution of the scorecard was less skewed than other wealth measures and not truncated, but demonstrated clumping. There was a higher level of agreement between the scorecard and the wealth index than consumption expenditure. The scorecard identified a similar number of poor households as the “basic needs” poverty line based on monthly consumption expenditure, with only 45 % errors of inclusion. However, it failed to pick up half of those living below the “basic needs” poverty line as being poor. Stakeholders supported the inclusion of water sources, income, food security and disability measures but had reservations about other items on the scorecard.

**Conclusion:**

In choosing poverty identification strategies for programmes seeking to enhance health equity it’s necessary to balance between community acceptability, local relevance and the need for such a strategy. It is important to ensure the strategy is efficient and less costly than alternatives in order to effectively reduce health disparities.

## Background

User fees gained widespread use as a means of alleviating pressure on constrained public health facility and district budgets in many sub-Saharan African countries and reducing unnecessary demand by populations [1, 2]. Yet user fees represent a financial burden for the poor and other vulnerable groups, and may reduce demand for health care services among these groups [3–5]. As a result, most countries have systems in place to exempt the poor from paying user fees in public facilities.

While it is relatively easy to identify patient groups (such as pregnant women), identifying the poor is more challenging. One commonly accepted method is to measure poverty through means testing [6, 7] based on income or consumption expenditure, with the latter being more reliable, less prone to seasonal variation and easier to collect in most rural settings [6, 8, 9]. However, in low income countries it can be difficult to measure individual income or consumption expenditure without undertaking costly surveys. The most common approaches to identify the poor for user fee exemptions include: proxy means testing, whereby a limited number of household or individual characteristics are used to judge poverty [10], the community or participatory wealth ranking method, whereby community members or leaders rank neighbouring households [11], and geographic targeting, whereby all residents in a given geographic area (e.g., village, district) are identified as poor (this is sometimes combined with community and proxy means testing) [12]. Geographical targeting is less precise relative to other methods used to target poor households, but is typically lower in cost and administratively simple [13, 14]. A lack of reliable methods to identify poor people in the community has affected implementation of exemption policies in low and middle income countries [15].

Previous studies have examined how these approaches compare to each other in terms of identifying the poor [16, 17]. One study considered the acceptability of community wealth ranking to identify the poor for a community health insurance scheme in Burkina Faso [18]. A study in Ghana compared means testing (based on consumption expenditure), proxy means testing (based on a wealth index) and the participatory wealth ranking approaches in terms of their classification of the poor [19]. Both proxy means testing and participatory ranking were found to have errors of inclusion, but participatory ranking was more acceptable to stakeholders. Another study compared these approaches in terms of their cost [19]. However, most existing studies considered these poverty measures hypothetically, rather than assessing actual measures being employed within exemption programmes. Understanding the reliability and acceptability of different approaches to poverty assessment is important to inform more effective exemption policies.

We compare the reliability of a proxy means testing approach being used to identify the poor within a pro-poor health insurance scheme in Tanzania (a scorecard - Table 1), relative to two conventional wealth measures (a wealth index and consumption expenditure), and the acceptability of the scorecard method among stakeholders at various levels in the country.

**Table 1:** Household Screening Form

0.1 Is there a woman residing in the household who has given birth during the previous 12 months?	Yes 01	If no, terminate interview
	No 02	
0.2 Does this woman currently have any insurance cover or has she had cover in the previous 12 months?	Yes 01	
	No 02	
0.3 Which kind of insurance does this woman have/did she have?	01 NHIF BIMA WAZAZI	Terminate interview if 01, 02, 03, 05, 06, 88
	02 NHIF EMPLOYER CONTRIBUTION	
	03 CHF/TIKA BIMA WAZAZI	
	04 CHF/TIKA SELF CONTRIBUTION	
	05 SHIB	
	06 PRIVATE INSURANCE	
	88 OTHER (SPECIFY)	
0.4 Interviewer instruction:		
Please score households from 1 to 3 depending on the nature of their house and their responses to the following questions.		
0.4a Nature of the house	01 Mud house with thatched roof	Score |__|__|
	02 Mud house with iron sheet roof	
	03 Brick house	
0.4b Distance to nearest health facility walking	01 60 minutes or more	|__|__|
	02 between 30 and 60 minutes	
	03 less than 30 minutes	
0.4c Water source	01 River/stream/lake	|__|__|
	02 Shared well or tap	
	03 Private well or tap	
0.4d Fuel for cooking	01 Firewood or animal dung or no facilities	|__|__|
	02Charcoal	
	03 Kerosene, gas or electricity	
0.4e Toilet facilities	01 Bush	|__|__|
	02 Shared latrine	
	03 Private latrine or toilet	
0.4f Daily income	01 Less than 1500 Tsh per day	|__|__|
	02 Between 1500–3000 Tsh per day	
	03 More than 3000 Tsh per day	
0.4 g Average number of meals per day	01 1 meal per day	|__|__|
	02 2 meals per day	
	03 3 meals per day	
0.4 h Number of children/vulnerable children in household	01 More than 4 children or one of your children is disabled or suffers from a chronic disease.	|__|__|
	02 3 or 4 children	
	03 Less than 3 children	
0.4i Total score for household	01 Score 8–13 = poor	|__|__|
ADD SCORES FROM 0.4a-0.4i		
	02 Score 14–18 = average	
	03 Score 19–24 = rich	

NB: Each component had three options, with a score ranging from one to three per an answer. Aggregation of the score made a total of 8 to 24 based on the score assigned on the observed/responded answer. Household scoring 8 – 16 qualified for the programme. In additional to the 8 questions households were asked about asset ownership, utilities and housing characteristics and total consumption expenditure in a typical month

Name of household head …………………………..

Name of woman who delivered in previous 12 months…………………….

If household is eligible and consent is obtained, continue with interview:

## Methods

### Study setting

User fees are charged at all public health facilities in Tanzania, but specific groups are exempt from their payment including: pregnant women, children under five years of age, people with chronic conditions, the elderly, and the poor. Households can enrol in the Community Health Fund (CHF), a community-based health insurance scheme, to avoid having to pay user fees at public primary care facilities, at an annual premium of between TZS 5,000 (US $ 2.40) to TZS 30,000 (US $ 14.40) depending on the district. The poor are also supposed to be eligible for free enrolment in the CHF. The National Health Insurance Fund (NHIF) also provides free access to services across all service levels and types of facility to its members. However, coverage is limited to the formal sector, and contributions are made directly at source (6 % of salary). National health insurance coverage is around 15 % [20].

There are various guidelines and commonly accepted practices in the Tanzanian health sector for identifying the poor, with the result that at the local level there is significant discretion for community leaders and health workers to determine which, if any, measures are to be applied [21, 22]. According to Mubyazi this policy failure to define ‘who are the poor’ or how the poor should be assessed has caused confusion among health-care providers [21]. Some of the people who are eligible for exemptions are still incurring out of pocket payments when seeking health care at public facilities [23], with variation across districts [4, 23].

In 2010 the Tanzania National Health Insurance Fund (NHIF), with technical support from GFA Consulting group and funds from the German Development Bank (KfW) introduced a programme to give poor pregnant women free NHIF health insurance cards in Mbeya and Tanga regions, and free CHF cards to their households. The scheme initially used individual targeting to identify poor women for enrolment in the scheme using a scorecard. It was expected that the scorecard system might subsequently be used for poverty identification elsewhere in the country. The scorecard is based on eight indicators (housing materials, roof type, sanitation, fuel for cooking, remoteness from health providers, income, food security (measured in terms of the number of meals per day) and the number of dependants and those with disabilities (Table 1). A score rate of between 8 and 16 points qualifies the person as eligible to receive the insurance card [24]. The poverty dimensions included on the scorecard were adapted from those used in voucher programmes elsewhere in Sub-Saharan Africa [25].

### Data collection methods

To assess the reliability of the poverty scorecard, a survey of 2,623 households of women who had delivered within the previous 12 months was conducted in April 2012 across two districts (Mbarali and Kilolo) as part of an evaluation of the free health insurance card programme [26]. Households were sampled using a modified version of the Expanded Program on Immunization (EPI) type sampling scheme from villages surrounding 48 health facilities within the districts [26]. Before the tool was administered, the household was screened using the NHIF poverty scorecard. The aim was to sample households of differing wealth status: two-thirds scoring poor or average and the remaining one-third non-poor and ineligible for the insurance card. Households were then asked a series of questions on ownership of assets and housing particulars and average monthly consumption expenditure in relation to food, clothing, housing services (rent), energy, transport, communication and spending on health (e.g., medicine, transport for health care seeking and consultation fees).

Purposive sampling was used to select 39 key informants for interviews across district, health facility and community levels. At the health facility level, three facilities were chosen to represent at least one facility at each level of the health system and based on geographic location in the district and annual caseload of deliveries. National poverty criteria used by the Tanzanian Social Action Fund (TASAF) were then reviewed with district managers to identify three of the poorest villages and corresponding hamlets in the catchment of each facility. Eight focus groups were conducted in these communities with three among men and six among women who had a child under the age of one year. The participants were asked about their impressions of the pro-poor health insurance program, including the criteria for identifying the poor. Interviews and focus groups were conducted by Tanzanian researchers in Swahili and digitally recorded. Interviewers used recordings and field notes to produce full transcripts which were translated into English. One of the researchers, who is fluent in Swahili and English, conducted quality checks on the transcripts and translations.

### Data analysis methods

We created a wealth index including indicators relating to housing characteristics (water source, toilet type, source of fuel used for cooking, source of light, nature of flooring, nature of roof, nature of walls, number of rooms) and assets (electricity, radio, TV, DVD player, mobile phone, iron, fridge, sewing machine, tables, sofas, cupboards, motorbike, car, bank account) using polychoric principal component analysis (PCA). Polychoric PCA is preferred since the standard PCA assumes a linear relationship between the variables [8] which does not apply well to dummy or categorical variables [27]. Further it has been argued that transforming categorical variables into dummy variable for use in PCA may lead to loss of potential information especially when the categories have a meaningful ordering [27]. Total monthly consumption expenditure was estimated as the sum of expenditure across the seven expenditure components identified above, in Tanzanian Shillings. When using the NHIF scorecard we estimated the overall score for each household.

We examined the distribution and probability density functions of each of these three measures (wealth index, consumption expenditure and NHIF scorecard) graphically to assess the degree of skewness, clumping and truncation by plotting histograms. Truncation can arise when there is little difference in measured values among households at the extreme of the distribution (e.g., poor and very poor). If an insufficient number of asset indicators are used, then households will be grouped (clumped) together in a small number of groups, which is also problematic for the generation of wealth groupings (e.g., terciles). To assess agreement of classification of households according to the three different measures, we derived terciles based on the wealth index, consumption expenditure and the NHIF score, and estimated Kappa statistics to assess the degree of agreement of classification between the three measures. The Kappa statistic is a measure of agreement between categorical variables, which takes into account the agreement expected by chance [28, 29], and a value of less than 0.5 is taken to indicate poor agreement between measures.

We also examined errors of exclusion and inclusion of households when using the NHIF scorecard as compared to the wealth index and consumption expenditure. Exclusion error is defined as the number of the poor households excluded over the total number of poor households. Whereas inclusion error is defined as the number of non-poor households identified as poor over the total number of households [30, 31]. When using a proxy means testing approach to poverty identification, an agreed threshold is needed to establish who is poor and who isn’t. When using consumption expenditure or income to measure wealth, the basic needs poverty line can be used to identify poor households, with all households falling below this line being considered as poor. The World Bank research routinely uses $1.25 per person per day as a benchmark to identify the poor below that figure [32]. When using a wealth index based on asset ownership information, for example, a relative wealth ranking is typically carried out, whereby those within the lowest quintile or tercile are considered to be poor. In principle, households could be classified according to the national distribution by drawing on Demographic and Health Survey data for example. In this study, we use the following approaches to determine who is poor for each wealth measure.

When considering household monthly consumption expenditure, we used the National Bureau of Statistics definition of basic needs poverty as consumption expenditure below 36,482 TZS per adult equivalent per month [33]. To estimate the total number of adult equivalents per household we used the following formula [34].

The equivalence scale used is

$$ {\mathrm{e}}_h = {\left({\mathrm{A}}_h + 0.5{\mathrm{K}}_h\right)}^{0.7}, $$

Where: A_*h*_ is the number of adults (above 15 years) in household *h* and K_*h*_ the number of children (0–15 years) in the household.

When considering the wealth index, we assumed that those in the lowest tercile were poor. Finally, when considering the NHIF scorecard, we assumed that those scoring in the range of 8–16 were poor, as this was the criterion for determining eligibility for the scheme.

After qualitative data collection, the researchers read all of the English transcripts for familiarisation. Thematic analysis was conducted by two researchers based on the poverty identification criteria. The two researchers each conducted coding of the interviews independently using NVIVO 9.0 (QSR International). Variation in coding between the two was discussed until consensus achieved. Responses across the different categories of respondent were compared using framework analysis.

## Results

### Household survey

Just under 90 % of the surveyed households were headed by males. The majority of household heads (87.6 %) were Christian (Table 2). Almost 80 % of the surveyed household heads had completed primary education, most (85.7 %) of the households were subsistence farmers, and only 2.9 % were enrolled in the CHF insurance scheme.

**Table 2:** Households Demographic Characteristics

Variable	All (*N* = 2,623)	
Characteristics of the Household head	*n*	%
Married	912	34.8
Gender is male	2,297	87.6
Religion		
Catholic	871	33.2
Other Christians	1,427	54.4
Muslim	251	9.57
Other	74	2.8
Education		
No school	264	10.1
Some primary education	189	7.2
Completed primary education/Some secondary education	2,054	78.3
Secondary education and above	114	4.4
Occupation		
Formal employment	82	3.1
Not working	102	3.9
Subsistence farmer	2,247	85.7
Self employed/small business	189	7.2
Insured by the CHF	2,623	2.9
	*n*	Mean [s.d]
Number of children under 5 in the household	2,623	2.85 [1.70]
Number of elderly over 65 in the household	2,623	0.05 [0.24]
Household size	2,623	4.89 [1.64]
Average age of household head	2,569	36.76 [11.64]
Wealth measures		
NHIF Scorecard – Index value	2,623	15.99 [2.23]
Monthly consumption expenditure in TZS	2,623	98,070.74 [85551.55]
Wealth index	2,623	−2.58e^−09^ [2.55]

### Distribution of wealth measures

**Fig. 1:**
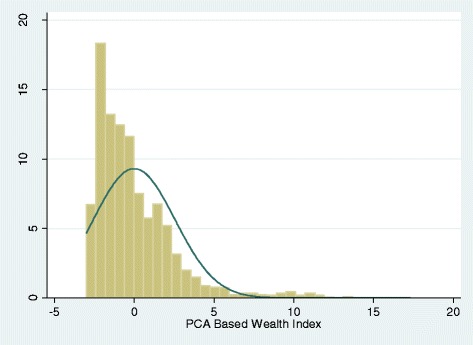
Wealth index derived using PCA. Distribution of the household wealth index scores created using principal component analysis (PCA) extraction method in Mbarali and Kilolo District: Tanzania

**Fig. 2:**
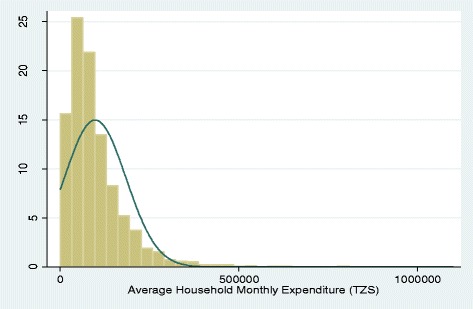
Reported average monthly consumption expenditure (TZS). Distribution of the household based on the reported consumption expenditure during the household survey in Mbarali and Kilolo District: Tanzania

**Fig. 3:**
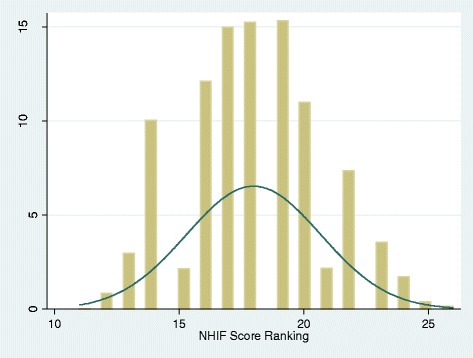
National Health Insurance Fund (NHIF) Scorecard. Distribution of the household based on the NHIF scorecard within the surveyed households in Mbarali and Kilolo District: Tanzania

The histograms based on the wealth index and consumption expenditure are skewed to the left, indicating that the majority of the sample are of lower wealth levels, with a more limited number of observations from higher wealth groups (Figs. 1 and 2). Both distributions also suffer from leftward truncation. In contrast, the histogram based on the scorecard was relatively normally distributed (Fig. 3). However, clumping was an issue for the scorecard, which may be explained by the smaller number of indicators used in its generation.

There was greater consistency in the overall classification of households by wealth category when comparing the wealth index and the NHIF scorecard (61 % classified in the same tercile), than consumption expenditure (43 % classified in the same tercile) (Table 3). The Kappa statistic was also higher indicating greater reliability between the wealth index and the scorecard, although in both cases the Kappa statistic remained below 0.50.

**Table 3:** Movement of households between terciles when comparing NHIF scorecard results to the wealth index and consumption expenditure

Movement of Households	Wealth index (PCA)	Consumption Expenditure
Proportion of households ranked in the same tercile	60.99	42.64
Proportion of households moving by one tercile	32.18	34.93
Proportion of households moving by two terciles	6.83	22.43
Kappa statistic	0.373***	0.122***

Statistically significant are *p < 0.05 **p < 0.01 ***p < 0.001

The basic needs poverty line identified the greatest number of poor households at just under 60 % (Table 4). A similar proportion of households were identified as poor by the scorecard, but a much lower level (33.36 %) were identified as poor by the wealth index (i.e., in the lowest tercile). Additional analysis showed that less than 33.36 % of our surveyed respondents fall into the lowest tercile in relation to the national Demographic and Health survey when comparing their wealth index values. About 68 % of the poor (basic needs poverty) were identified by the scorecard, compared to over 91 % of those in the bottom tercile of the wealth index. The scorecard resulted in errors of exclusion of around 32 % and 9 % with respect to the basic needs poverty line and the lowest wealth tercile respectively; while the error of inclusion was 45 % with respect to the basic needs poverty line, and 42 % with respect to the lowest wealth tercile.

**Table 4:** Identification of the poor and errors of inclusion and exclusion

	All (*n* = 2,623)	
Identification Mechanism	*n*	%
Wealth index (PCA) (bottom tertile)	875	33.36
Consumption Expenditure (CE) (below “basic needs” poverty line)	1,540	58.71
Scorecard (grade between 8–16)	1,534	58.48
Among those below the national basic needs poverty line	(*n* = 1540)	
Identified as poor by the scorecard	1,049	68.12
Identified as poor by the wealth index	643	41.75
Among those in the lowest wealth tercile	(*n* = 875)	
Identified as poor by the scorecard	795	90.86
In relation to the basic needs poverty line		
Errors of inclusion (% of those above the basic needs poverty classified as poor by scorecard)	485	44.78
Errors of exclusion (% of those below basic needs poverty not classified as poor by scorecard)	491	31.88
In relation to the wealth index		
Errors of inclusion (% classified as poor by the scorecard who are not in lowest tercile)	739	42.27
Errors of exclusion (% of those in lowest tercile not classified as poor by scorecard)	80	9.14

### Key informant interviews and FGDs

Among the key informants, 48 % were female and 52 % male. For government key informants (including health providers), total years of service in the government ranged from 4 to 30 years with a range of 2 to 12 years in their current position. Respondents from non-governmental organizations had been in their current roles approximately 4 to 5 years. The age of women participating in the focus groups ranged from 22 to 45 years old, with a similar range among men (24 to 46 years old).

There was a notably high level of consensus among stakeholders across different levels regarding the poverty criteria. Although positive about the overall aims of the program, respondents exhibited scepticism about the poverty targeting criteria in the scorecard and emphasized the general conditions of poverty throughout their context. Nonetheless, there was general consensus that water sources, daily income, and average number of meals per day were good criteria for identifying the poor in their context.

*“..…For a pregnant woman to eat one meal is not right. She is supposed to eat more for the health of the mother and growth of the baby, but when she eats one meal it means she has nothing to eat. She thinks, ‘if I eat three times today, what about tomorrow?”’* (Male FGD participant)

*“…Women walk long distances to fetch shallow water in the rivers and streams despite some of the villages having community tap water. But still, women spend a lot of time to get water”* (Health provider)

*“….Money is everything. If the woman is selling doughnuts and she gets very little profit ….. and she is craving meat then she cannot even afford to buy it.”* (Female FGD participant)

Participants described various reasons behind their perceptions of the score card criteria. For the criteria related to housing materials, respondents indicated that this may be appropriate but could also reflect individual or cultural preferences rather than poverty. Given a certain amount of resources, they felt that people will make different decisions that might not align with these criteria. Others, such as the presence of a toilet, were considered a poor indicator of poverty and instead an indicator of knowledge regarding sanitation and hygiene.

“....*One can have a nice house with iron sheets, but they do not have food to eat, maybe they inherited the house from their forefathers. Others they have mud and thatch houses but they have plenty of cattle and more food in storage*.” (Female FGD participant)

*“…some of the families do not think having a better house is a priority. They give priority to other things like education. Others live in a thatched house and when you look, their standards of living are better - eating well, dressing well and doing all things well - but they do not take all the children to school*.“(Community leader)

*“.....Regarding the toilet, it’s not a criterion for poverty at all, it’s all about health of the people and ignorance of the people. For the toilet even if you are poor, [you] cannot fail to dig a pit latrine,”* (Male FGD participant)

When discussing the indicator distance to a health facility, many of the respondents felt that this was not useful for individual level targeting due to the fact that decisions about where to build facilities are determined by government actors. In addition, in some settings, there are poor women living very close to the facilities.

*“I think, she can be living far or near and still be poor.”* (Health provider)

*“…but we should not say she is poor on her own. It is national poverty because they could build health facility every 30 kilometres as the policy says that there should be health facility in each village”* (District respondent)

There were divergent opinions about whether the number of children should be considered in determining a woman’s poverty status which did not appear to follow patterns based on type of respondent. Some respondents recognized that additional children might put a strain on available resources, while others noted that some families might have many children and yet be in a good position financially. Responses regarding children with disabilities followed a similar pattern. For both, respondents indicated that it would need to be considered in light of other factors.

*“…It can be true or not true, because someone can have many children - more than six - but have a good environment. Another one might have two children or one, but fail to take care [of] the family…”* (Influential Female Community Member)

*“If you have many children, you have to spend a lot so expenditures are high. So that can cause the family to be poor and fail even to pay for the health services.” (*District manager)

*“It will depend, because what are the causes of the disability? The experts they can say that maybe the disability was caused by lacking basic things…But disability as disability cannot cause poverty. For those who pray they say that is just God’s wish, he has seen the creation should be this way. (Community leader)*

*“[having a child with a disability] also can contribute, because you can have additional burdens. You can even fail to do other tasks because you are caring for this child.”* (Health Provider)

When asked about additional criteria to consider, a number of respondents mentioned widows and young women citing that they will have less access to resources because of their status. They noted that some criteria such as clothing were important indicators likely to result in discrimination at the health facility. Whether children are attending school was also mentioned by a number of respondents.

*“If a teacher or the wife of one of the civil servants because she dresses properly and you go to the dispensary and you are pregnant, it is discouraging because we stay a very long time waiting for the services, whereas they just call her and leave you behind.”* (Female FGD participant)

*“Even though not all those who don’t go to school are poor, for primary education, every parent would like his or her child to at least be able to write and read. She or he may fail to take his or her child to secondary school, but not primary education.* (District manager)

Issues of how many cows they have, what land they have for farming, and whether they have money in the bank were also raised as potential indicators of poverty.

When asked their views about how to identify the poor, FGD respondents tended to say that the local government representatives had the ability to identify the poor. Yet, a number of respondents also expressed a lack of confidence in village leaders to fairly identify the poor. Some indicated concern about bias from the village leaders. One FGD respondent described how this had recently occurred in a maize distribution program for the poor. Another described how the reliability of these leaders varies across villages – with some providing aid when needed and others routinely avoiding work.

## Discussion

The identification and targeting of the poor in relation to health programmes is one approach being used by policy makers to promote equity in health coverage. However, for such an approach to be effective in reaching its goal of enhancing health equity, it relies on the accurate assessment of poverty and identification of the poor. While there have been numerous studies comparing different approaches to poverty measurement, there have been few evaluating mechanisms used to identify the poor in health programmes, with some exceptions [18]. This paper set out to compare the poverty scorecard method used to identify households eligible for a free health insurance card to enhance maternal health care access, relative to two conventional methods of individual wealth ranking, a wealth index and monthly consumption expenditure, and to assess the acceptability of the scorecard method to key stakeholders.

The scorecard technique, when used to rank households, produced a distribution that was less skewed than other wealth measures and not truncated. However, the distribution demonstrated more clumping than the other measures of wealth, which may reduce its ability to differentiate between poor and very poor households. There was greater consistency in the overall classification of households by wealth between the wealth index and the scorecard method, than consumption expenditure, although the Kappa statistic was lower than 0.5 in both cases.

The scorecard identified a similar number of poor households to the basic needs poverty line defined in terms of a threshold of monthly consumption expenditure per adult equivalent, with only 45 % being errors of inclusion. However, it failed to pick up a third of those living below the basic needs poverty line as being poor, meaning that a large proportion of the poor would not have been considered eligible for the free health insurance programme.

Figures were similar when comparing the scorecard to a poverty threshold determined by the wealth index (the bottom wealth tercile), although errors of inclusion were higher, as fewer individuals were classified as poor when using the wealth index threshold. We found a much lower level of errors of inclusion, and similar levels of errors of exclusion to that reported elsewhere [30]. This demonstrates the ability of proxy means testing to yield better results in different settings, a finding also illustrated previously [30], especially when the wealth proxies are locally determined and deemed relevant. Indeed, the degree of agreement between different approaches to poverty identification has been found to vary by context [30]. However, we were unable to examine the effect of context in this study as our study sites were fairly homogenous and rural.

Among stakeholders interviewed, there was general consensus that water sources, daily income, and average number of meals per day were good criteria for identifying the poor in their context. Food security, asset ownership, and disability have also been identified as important determinants of poverty in participatory wealth ranking exercises elsewhere (e.g., [18, 19, 35]). However, stakeholders expressed scepticism about the ability of other indicators to reliably identify the poor (sanitation, housing materials, roof type, number of children, and distance from the health facility), indicating that these might be the result of individual choices or structural factors beyond their control.

Household characteristics identified as important in other studies were not included in the scorecard, such as social exclusion [18, 19, 35], age [18], appearance [19], land and livestock ownership, and being childless [35]; however, these criteria, other than appearance, were not mentioned by stakeholders interviewed in this study as being relevant.

## Conclusion

When choosing poverty identification strategies for health programmes aimed at enhancing equity, community acceptability and local relevance must be balanced against the need for a reliable and efficient strategy that can be employed easily and at minimum cost [36]. A proxy means testing approach (a scorecard) used to identify poor households for free health insurance can be effective in identifying the poor, increasing the affordability of health care among this group, but consideration of the implementation process surrounding a given poverty identification strategy should also be examined, as this may vary by context and individual targeting may be open to misuse. Further examination of this issue would be an important area for future research.

## Abbreviations

CE: Consumption expenditure
CHF: Community Health Fund
FGD: Focus Group Discussion
KfW: German Development Bank
NHIF: National Health Insurance Fund
PCA: Principal component analysis
TZS: Tanzanian shilling (US $ 1 == 2084 TZS)
